# A Retrospective Study on the Expression of E-Cadherin in Endometrial Carcinoma

**DOI:** 10.7759/cureus.70767

**Published:** 2024-10-03

**Authors:** MonishaRita Jayaraman, Niveditha EN, Lakshmipriya V, Volga Harikrishnan

**Affiliations:** 1 Pathology, Saveetha Medical College and Hospital, Saveetha Institute of Medical and Technical Sciences, Saveetha University, Chennai, IND

**Keywords:** cancer metastasis, dedifferentiated carcinoma, e-cadherin expression, endometrial carcinoma, endometrioid carcinoma, serous carcinoma

## Abstract

E-cadherin is a cell adhesion molecule crucial for maintaining cellular integrity and survival. E-cadherin is the focus of this study, which aims to evaluate its immunohistochemical staining patterns in endometrial carcinoma (EC).

Material and methods

In this retrospective study, 25 formalin-fixed, paraffin-embedded tissue blocks of EC were retrieved from the Department of Pathology at Saveetha Medical College. These samples, collected between September 2020 and May 2023, were identified using unique histopathology numbers.

Results

The analysis revealed that E-cadherin expression decreased as the endometrial tissue progressed from normal to carcinoma. This reduction was more pronounced in cancers with greater depth of invasion and in more advanced stages compared to those with less invasive depth and earlier stages. Additionally, E-cadherin expression was higher in grade 1 tumors but became heterogeneous or negative in grade 3 tumors.

Conclusion

The downregulation of E-cadherin is likely due to disruptions in the cadherin-catenin complex, which is linked to the potential for local and distant metastasis in cancer. This study underscores the significance of cell adhesion molecule expression in predicting the prognosis of EC.

## Introduction

Endometrial carcinoma (EC) is the most common cancer of the female reproductive system, primarily affecting the lining of the uterus, known as the endometrium [1]. It typically occurs in postmenopausal women, with the majority of cases diagnosed in women over the age of 50 [1]. The most significant risk factors include obesity, hypertension, diabetes, and unopposed estrogen exposure, such as that seen with hormone replacement therapy or conditions such as polycystic ovary syndrome (PCOS) [1]. E-cadherin is a crucial cell adhesion molecule that plays a vital role in maintaining tissue architecture and cellular integrity by mediating cell-cell adhesion in epithelial tissues [2]. It is involved in various physiological processes, including embryogenesis and wound healing. E-cadherin functions as a tumor suppressor, and its downregulation or loss is often associated with cancer progression, leading to increased cell motility, invasion, and metastasis [2]. In cancers such as EC, reduced E-cadherin expression correlates with more aggressive tumor behavior and poorer prognosis, highlighting its importance in both normal tissue function and disease.

## Materials and methods

Relevant clinical information, including the patient's age, clinical symptoms, parity, and endometrial thickness for the biopsy samples, was gathered from the requisition forms provided by the clinician. For total abdominal hysterectomy specimens, the gross measurements of endometrial thickness were recorded. Tissue samples fixed in 10% neutral buffered formalin were retrieved and trimmed from paraffin blocks.

Sections measuring three to four microns were prepared from each tissue block using a Leica 2125RTS manual rotary microtome. These sections were mounted on labeled glass slides coated with egg albumin, air-dried, and placed in a staining rack. They were then stained with Harris hematoxylin and eosin using a Leica Auto Stainer XL (ST5010). Hematoxylin- and eosin-stained slides were examined to assess the adequacy of tumor components. Blocks with sufficient material were selected for further analysis. Once dry, the slides were stored according to study numbers and reviewed by the principal investigator, who recorded the findings on a data sheet.

For immunohistochemistry, sections of two to four microns were cut from each block and mounted on poly-L-lysine treated slides, labeled with the lab number and test type. After drying, these slides were stored and processed in batches. Each batch included one positive and one negative control subjected to the same testing conditions. Manual immunostaining was performed, using E-cadherin rabbit monoclonal antibody clone EP6 as the primary antibody. The secondary antibody used was the Poly Excel HRP/DAB detection system, a two-step universal kit for both mouse and rabbit primary antibodies.

Immunohistochemical results were interpreted following the methodology of a previous study [3,4]. At least 10 low-power fields (10x) were examined per case. Two pathologists independently analyzed the stained slides to minimize inter-observer variation. The evaluation focused on the presence of staining (indicated by brownish coloration), the cellular localization (nuclear/membrane/cytoplasm), and the percentage of cells stained. Strong intermembranous staining of E-cadherin without cytoplasmic positivity was classified as positive. Aberrant staining patterns, such as weak or discontinuous membranous staining, membrano-cytoplasmic staining, or perinuclear dot-like staining, were classified as negative.

The scoring system, as detailed in Table 1, was as follows: a score of 3 was assigned if more than 75% of cells stained positive, 2 if 51-75% stained positive, 1 if 6-50% stained positive, and 0 if fewer than 5% stained positive. A score of 3 indicated normal E-cadherin expression, while scores of 1 and 2 indicated heterogeneous expression due to downregulation. A score of 0 indicated a complete loss of E-cadherin expression.

**Table 1:** Interpretation of the immunohistochemical staining pattern of E-cadherin

Percentage of cells positive	Score	Staining pattern	E-cadherin expression
>75	3	Homogenous	Normally expressed
51-75	2	Heterogenous	Downregulated
6-50	1	Heterogenous	Downregulated
<5	0	Negative	Absent

## Results

**Figure 1:**
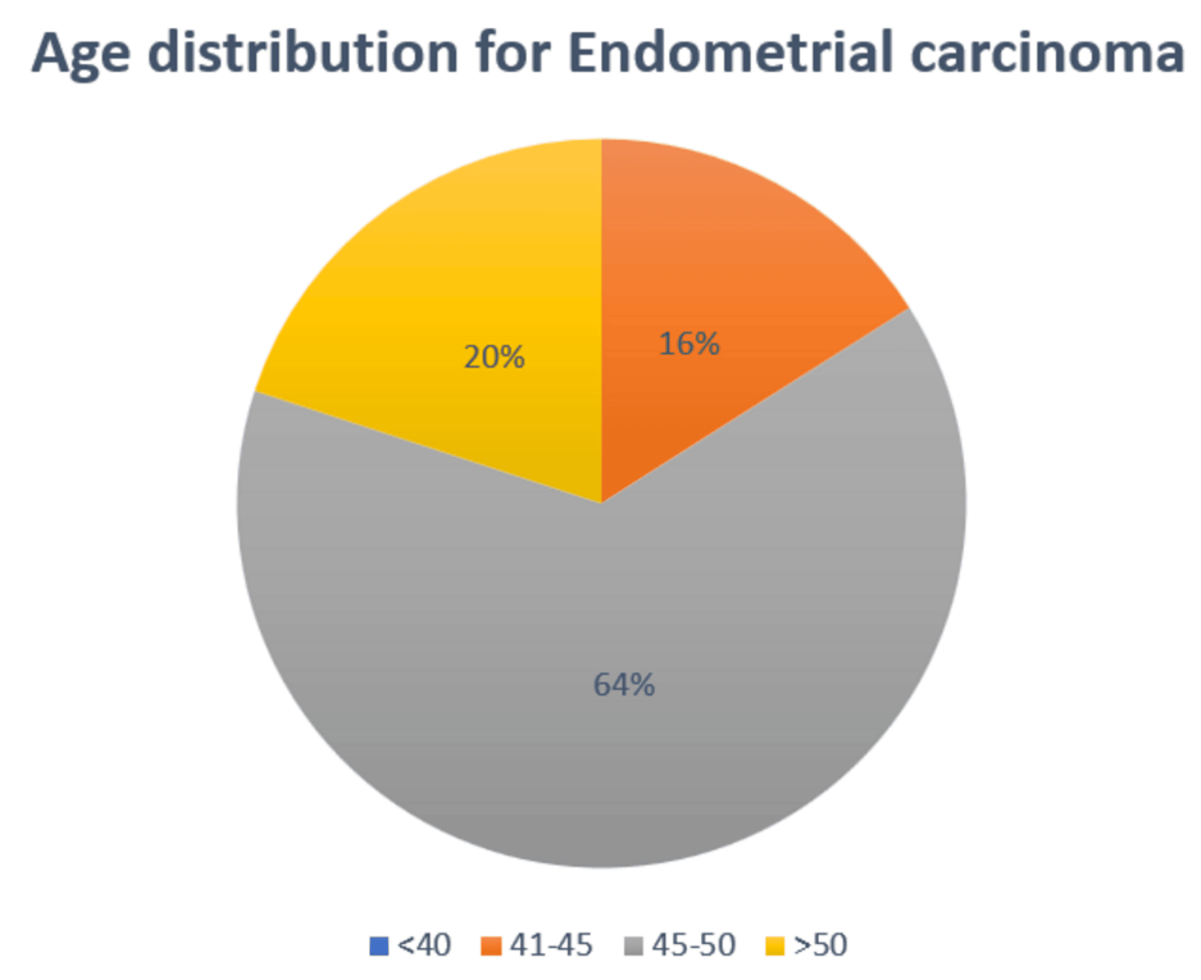
Age-wise distribution of the cases of endometrial carcinoma

The study involved 25 cases of EC. Age is a risk factor in cases of malignant transformation of EH without atypia and AH into EC as the change occurs over time with age. The majority of the cases of EC fell under the age group of 41-50 years which is represented in Figure 1.

**Figure 2:**
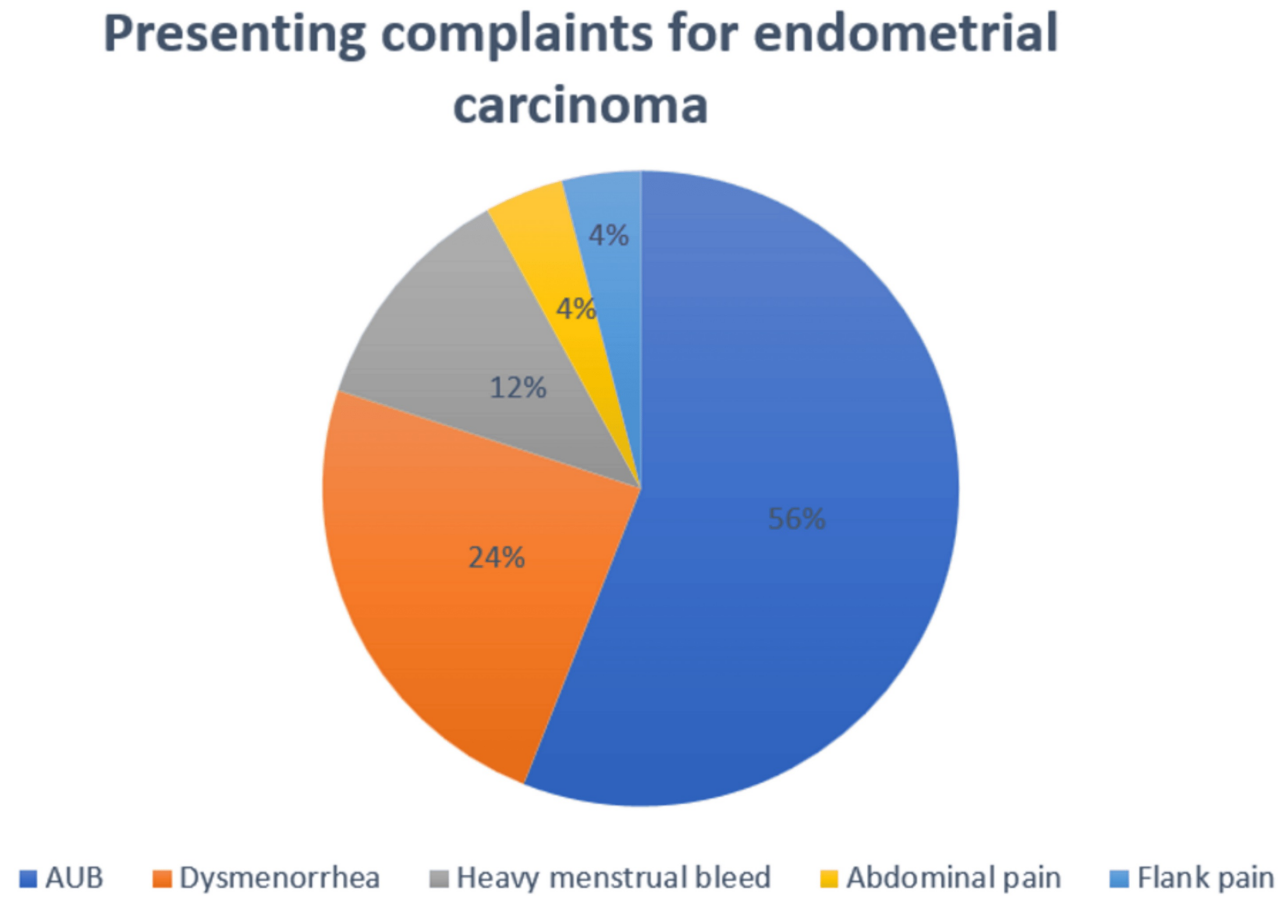
Distribution of presenting symptoms of endometrial carcinoma

The most common symptom at presentation for EC was bleeding per vagina. Many of the cases presented with bleeding per vagina, heavy menstrual bleeding, abnormal uterine bleeding and abdominal pain. Thirty-six percent of the cases had abnormal uterine bleeding as the presenting complaint, which is a typical presentation of EC given the age of incidence. The second most frequent complaint at presentation is bleeding per vaginum. This is recorded in peri menopausal women of all age groups. Vague symptoms such as flank pain also have been the initial presenting complaint in a few cases. The pictorial representation of the data is given in Figure 2.

The endometrial thickness in patients with carcinoma endometrium were compared. The cases were arbitrarily divided into two groups of endometrial thickness more than 10 mm and endometrial thickness less than 10 mm. Around 84% of the patients with EC had endometrial thickness more than 10 mm. The endometrial thickness in EC is shown in Table 2.

**Table 2:** Endometrial thickness in cases of endometrial carcinoma

Endometrial thickness in cm	Frequency	Percentage
≤10	4	16.0
>10	21	84.0
Total	25	100.0

Among the 25 cases of carcinoma, 20 of them are the histologic type endometrioid carcinoma (EEC), not otherwise specified (NOS). The remaining cases are four serous EC (ESC) and one case of dedifferentiated carcinoma (DDC). The distribution is showed in Table 3.

**Table 3:** Various histologic variants of endometrial carcinoma included in the study NOS: not otherwise specified

Histological type	Frequency	Percentage
Endometrioid Carcinoma NOS	20	80.0
Serous Carcinoma	4	16.0
Dedifferentiated Carcinoma	1	4.0
Total	25	100.0

**Figure 3:**
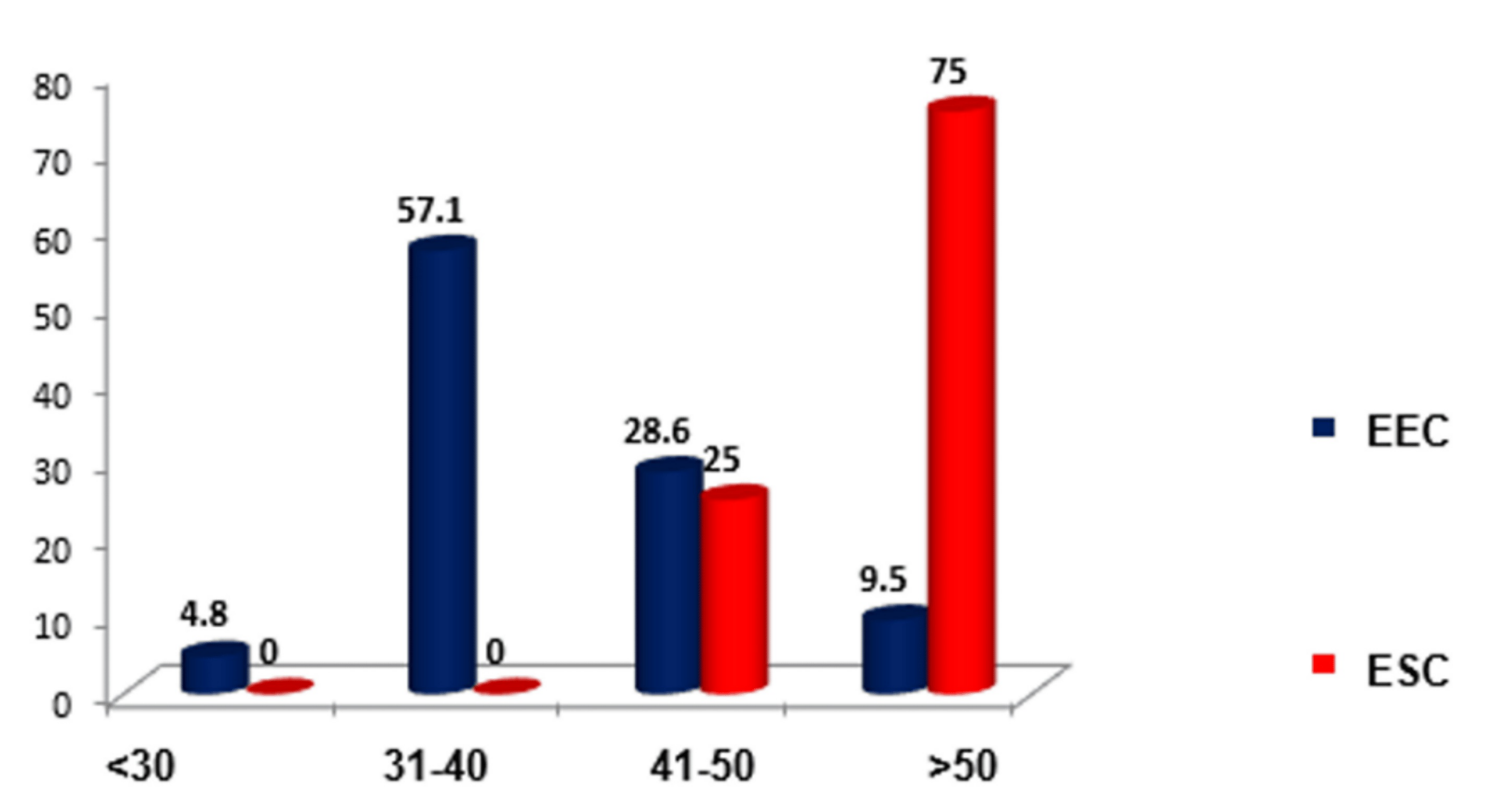
Age distribution in various types of endometrial carcinoma EEC: endometrial endometrioid carcinoma, ESC: endometrial serous carcinoma

The incidence of serous carcinoma is in older age group of women, whereas the incidence of endometrioid carcinoma is in a relatively younger age group, which is shown in Figure 3.

**Figure 4:**
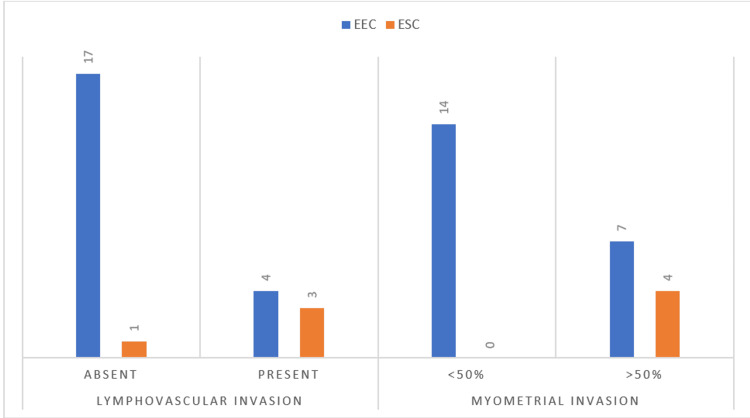
Number of cases showing lymphovascular invasion and myometrial invasion EEC: endometrial endometrioid carcinoma, ESC: endometrial serous carcinoma

All the cases of serous carcinoma showed myometrial invasion more than 50%, whereas only 33% of the cases of endometrioid carcinoma showed more than 50% of myometrial invasion. Additionally, 75% of the cases of serous carcinoma showed lymphovascular invasion, and only 19 percent of endometrioid carcinoma showed lymphovascular invasion (LVI). Serous carcinoma is more prone to myometrial invasion of more than 50% of the myometrium than compared to endometrioid carcinoma. This can be attributed to the fact that serous carcinomas are more aggressive compared to endometrioid carcinomas. Serous carcinoma also shows more propensity to show lymphovascular invasion in comparison to endometrioid carcinoma. Seventy-five percent of the cases of serous carcinoma showed evidence of lymphovascular invasion, whereas only 19% of endometrioid carcinomas showed lymphovascular invasion. The data are illustrated in Figure 4.

The comparison of E-cadherin expression between endometrioid carcinoma and serous carcinoma, using Fisher's exact test, yielded a p-value greater than 0.05, indicating no significant difference. This analysis suggests that there is no correlation between histologic type and E-cadherin expression. When comparing E-cadherin scores between the two carcinoma types, Fisher's exact test resulted in a p-value of 0.142, which is not statistically significant. Furthermore, an assessment of the correlation between E-cadherin expression and its downregulation produced a p-value of 0.275, also indicating no significant correlation. The statistical comparison of E-cadherin expression in endometrioid versus serous carcinoma is summarized in Table 4, showing no significant correlation between E-cadherin downregulation and the histologic type of EC.

**Table 4:** Comparison of E-cadherin score in endometrial endometrioid carcinoma and endometrial serous carcinoma *p<0.05, ***p<0.001: statistically significant, ns: not significant, NOS: not otherwise specified

Parameter	Endometrioid Carcinoma NOS N (%)	Serous Carcinoma N (%)	Total N (%)	X^2^ – Value	p value
E - cadherin - Positive score categorization				3.183	0.142 (ns)
Negative	09 (42.9)	04 (100)	13 (52)		
Heterogeneous	07 (33.3)	0	07 (28)		
Normal	05 (23.8)	0	05 (20)		
Total	21 (100)	04 (100)	25 (100)		
E- cadherin –Expression					
Normal	05 (23.8)	0	05 (20)	1.190	0.275 (ns)
Down regulated	16 (76.2)	04 (100)	20 (80)		
Total	21 (100)	04 (100)	25 (100)		

The relationship between E-cadherin expression and histologic grade in EC was analyzed using Fisher's exact test, yielding a p-value of 0.05, which is considered significant. This result indicates that E-cadherin expression correlates with the histologic grade of the tumor, demonstrating that E-cadherin levels decrease as the tumor grade advances. Detailed statistical data on E-cadherin expression across different tumor grades show a marked reduction in expression in tumors with higher grades. Additionally, E-cadherin expression was evaluated in relation to myometrial invasion, with a p-value of 0.025, indicating statistical significance. This correlation was assessed using both the International Federation of Gynecology and Obstetrics (FIGO) and TNM staging systems, both of which revealed significant results. These findings suggest that E-cadherin expression decreases with increasing tumor stage. Statistical details on E-cadherin expression across various stages of carcinoma, according to both FIGO and TNM classifications, are presented in Table 5.

**Table 5:** Comparison of E-cadherin expression across various stages of carcinoma, according to both FIGO and TNM classifications *p<0.05, ***p<0.001: statistically significant, ns: not significant, FIGO: International Federation of Gynecology and Obstetrics

Study Parameter	E-cadherin marker - Positive score categorization			Total N (%)	X^2^-Value	p value
	Negative N (%)	Heterogeneous N (%)	Normal N (%)			
Histological Grade						
Grade I	02 (15.4)	04 (57.1)	03 (60)	09 (36)	7.912	0.05*
Grade II	05 (38.5)	03 (42.9)	02 (40)	10 (40)		
Grade III	06 (46.2)	0	0	06 (24)		
Total	13 (100)	07 (100)	05 (100)	25 (100)		
Myometrial invasion						
<50	04 (30.8)	05 (71.4)	05 (100)	14 (56)	7.611	0.025**
>50	09 (69.2)	02 (28.6)	0	11 (44)		
Total	13 (100)	07 (100)	05 (100)	25 (100)		
FIGO stage						
Ia,Ib,II	04 (30.8)	06 (85.7)	05 (100)	15 (60)	9.241	0.008***
IIIa, IIIb	09 (69.2)	01 (14.3)	0	10 (40)		
Total	13 (100	07 (100)	05 (100)	25 (100)		
TNM Stage						
T1& T2	03 (23.1)	06 (85.7)	05 (100)	14 (56)	11.641	0.002***
T3	10 (76.9)	01 (14.3)	0	11 (44)		
Total	13 (100	07 (100)	05 (100)	25 (100)		

**Figure 5:**
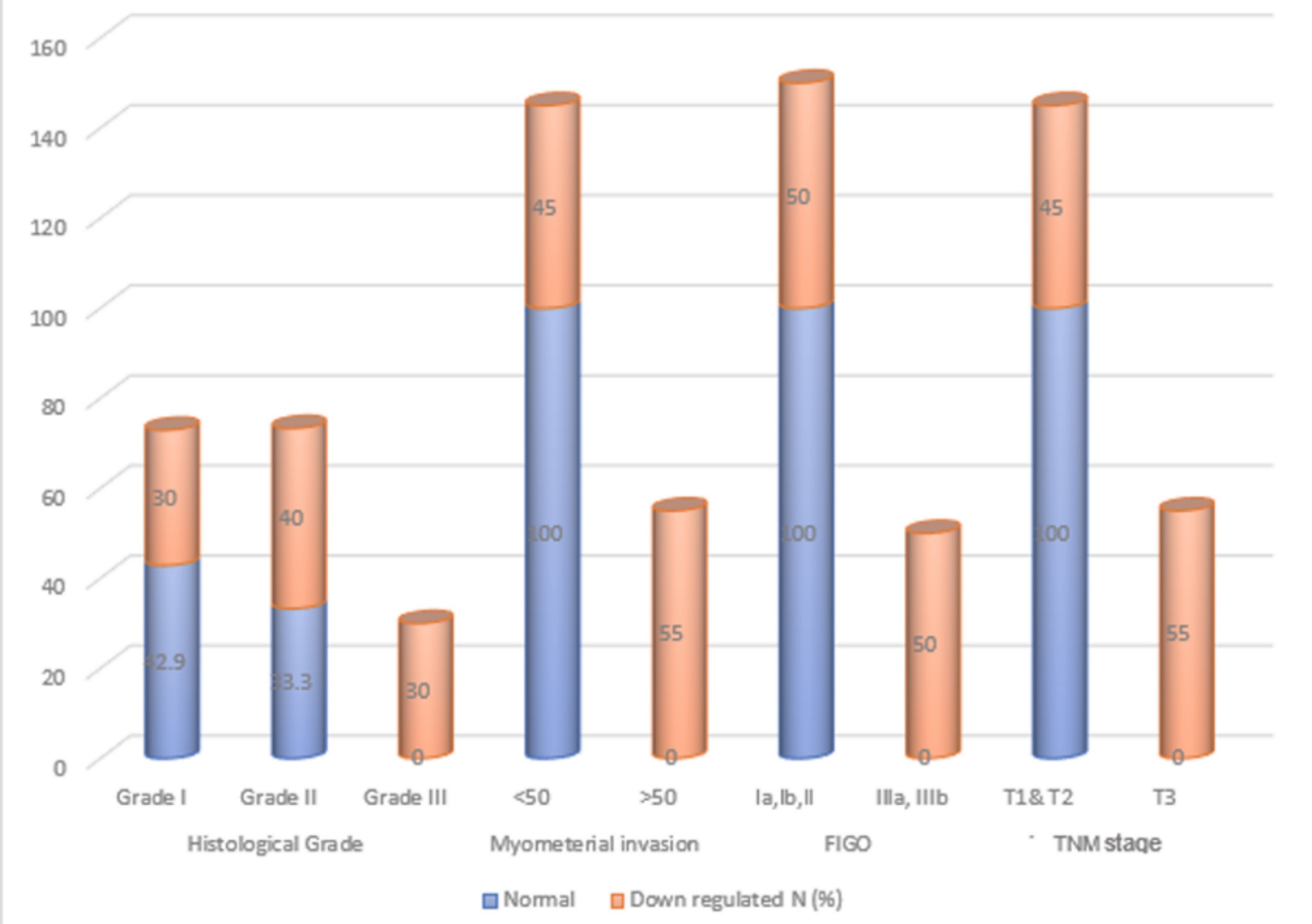
Comparison of E-cadherin expression across tumour grade, stage, and myometrial invasion

The comparison of tumor grade, myometrial invasion, and stage of tumor is shown in Figure 5. In grade 1 tumors, there is downregulation of E-cadherin in 30% of the cases, whereas it is 40% in grade 2 tumors. However, when it comes to grade 3, all the cases showed downregulation of E-cadherin. In tumors with myometrial invasion less than 50%, only 45% of the cases demonstrated decreased expression of E-cadherin however in tumours having more than 50% myometrial invasion, all the cases showed downregulation of E-cadherin. Figure 5 shows a pictorial representation of the data comparing tumour grade, stage and myometrial invasion with E-cadherin expression.

The expression of E-cadherin score is compared with lymph node status, lymphovascular invasion, and depth of invasion using Fisher's exact test. The p-value for these were 0.123, 0.752, and 0.123, respectively. None of these factors showed significant corelation to the expression of E-cadherin. The statistical analysis and p-value calculation is shown in Table 6.

**Table 6:** Comparison of expression of E-cadherin with lymph node status, lymphovascular invasion, and depth of invasion *p<0.05, ***p<0.001: statistically significant, ns: not significant

Study Parameter	E cadherin marker - Positive score categorization			Total N (%)	X^2^ -Value	p value
	Negative N (%)	Heterogeneous N (%)	Normal N (%)			
Lymph node status						
Negative	07 (53.8)	06 (85.7)	05 (100)	18 (72)	4.004	0.123 (ns)
Positive	06 (46.2)	01 (14.3)	0	07 (28)		
Total	13 (100)	07 (100)	05 (100)	25 (100)		
Lymphovascular invasion						
Absent	07 (53.8)	04 (57.1)	04 (80)	15 (60)	0.52	0.752 (ns)
Present	06 (46.2)	03 (42.9)	01 (20)	10 (40)		
Total	13 (100)	07 (100)	05 (100)	25 (100)		
Depth of invasion						
≤10	07 (53.8)	06 (85.7)	05 (100)	18 (72)	4.004	0.123 (ns)
>10	06 (46.2)	01 (14.3)	0	07 (28)		
Total	13 (100)	07 (100)	05 (100)	25 (100)		

**Figure 6:**
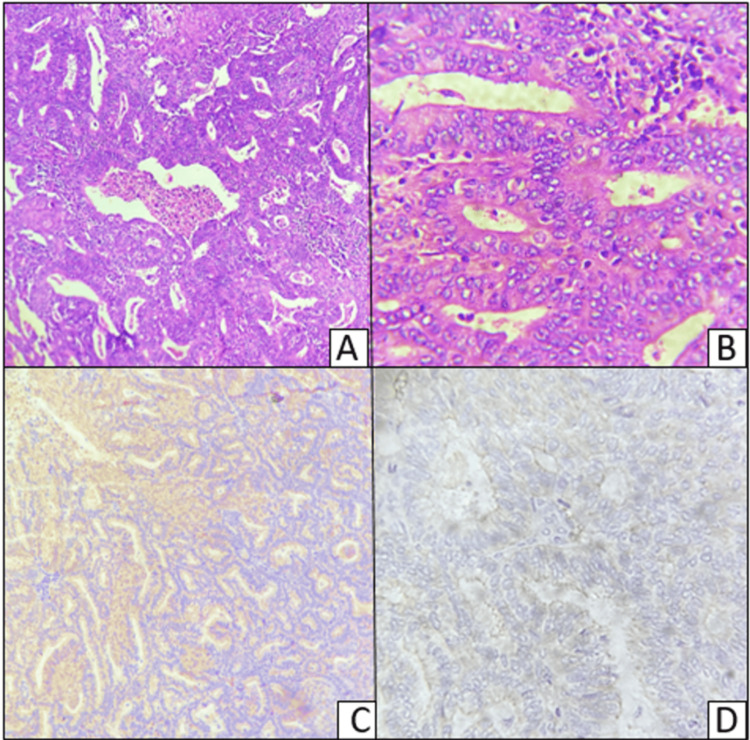
A,B - Hematoxylin and eosin-stained sections and C,D - immunosuhistochemical staining of endometrial endometrioid carcinoma A (H&E 100X) and B (H&E 400X) show endometrial carcinoma; C (E-cadherin, 100X) shows cytoplasmic positive staining to the right side and negative staining to the left; D (E-cadherin, 400X) shows negative staining and focal weak membrano-cytoplasmic staining of the endometrial glands

Figure 6 shows a hematoxylin and eosin-stained image of endometrial endometrioid carcinoma and the corresponding section stained with E-cadherin. The section shows back-to-back arrangement of endometrial glands with no intervening stroma, typical of endometrioid carcinoma, and immunohistochemistry shows area with positive cytoplasmic staining for E-cadherin and a focal negative area.

**Figure 7:**
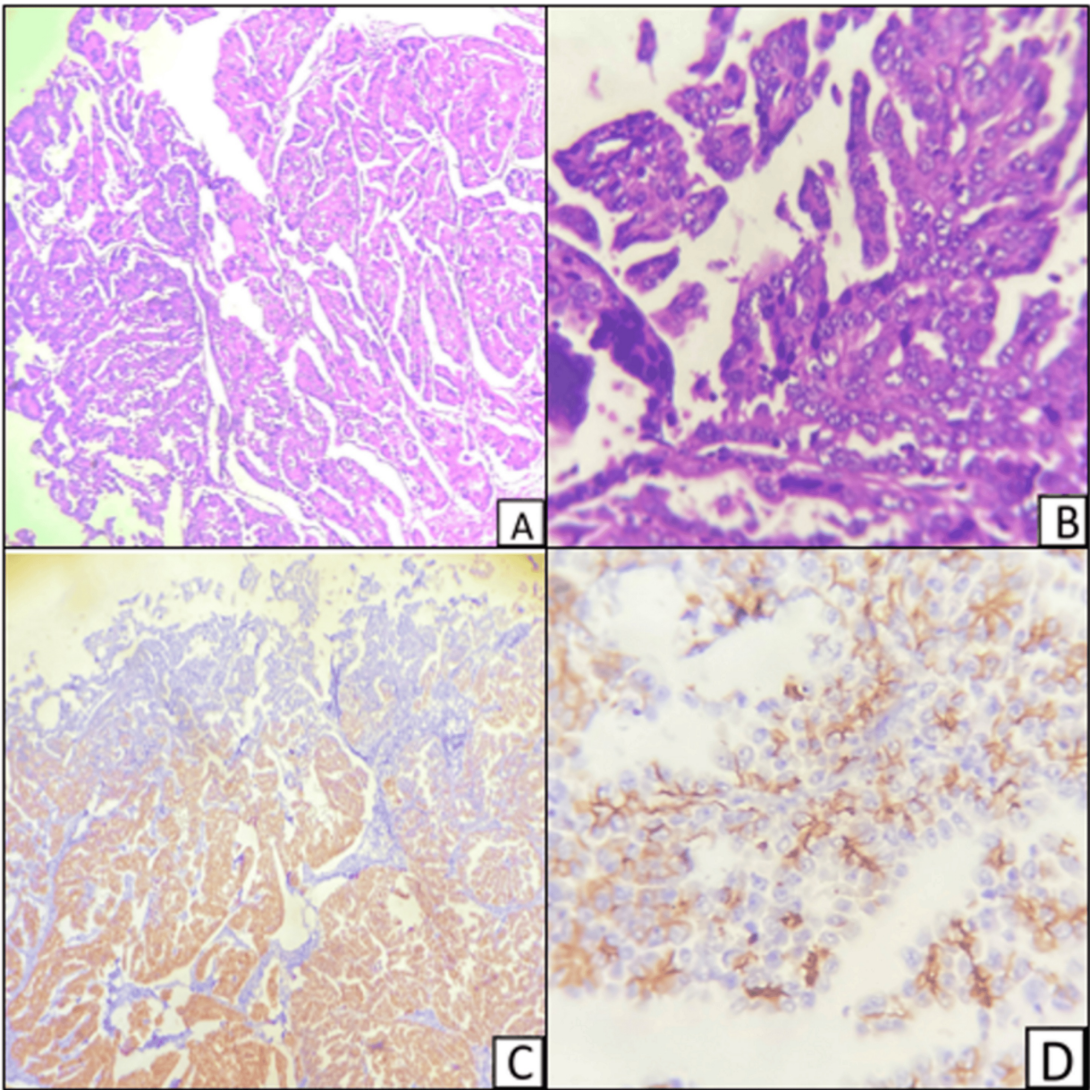
A,B - Hematoxylin and eosin-stained sections; C,D - immunohistochemical-stained section of endometrial serous carcinoma A (H&E 100X) and B (H&E 400X) show serous carcinoma of the endometrium; C (E-cadherin, 100X) shows membrano-cytoplasmic positive staining in the bottom and negative staining to the top; D (E-cadherin, 400X) shows negative staining and focal incomplete membrano-cytoplasmic staining of the endometrial glands

Figure 7 shows serous carcinoma of the endometrium showing the typical papillary architecture and pleomorphic nuclei. The corresponding sections stained for E-cadherin show negative staining and focal incomplete staining.

**Figure 8:**
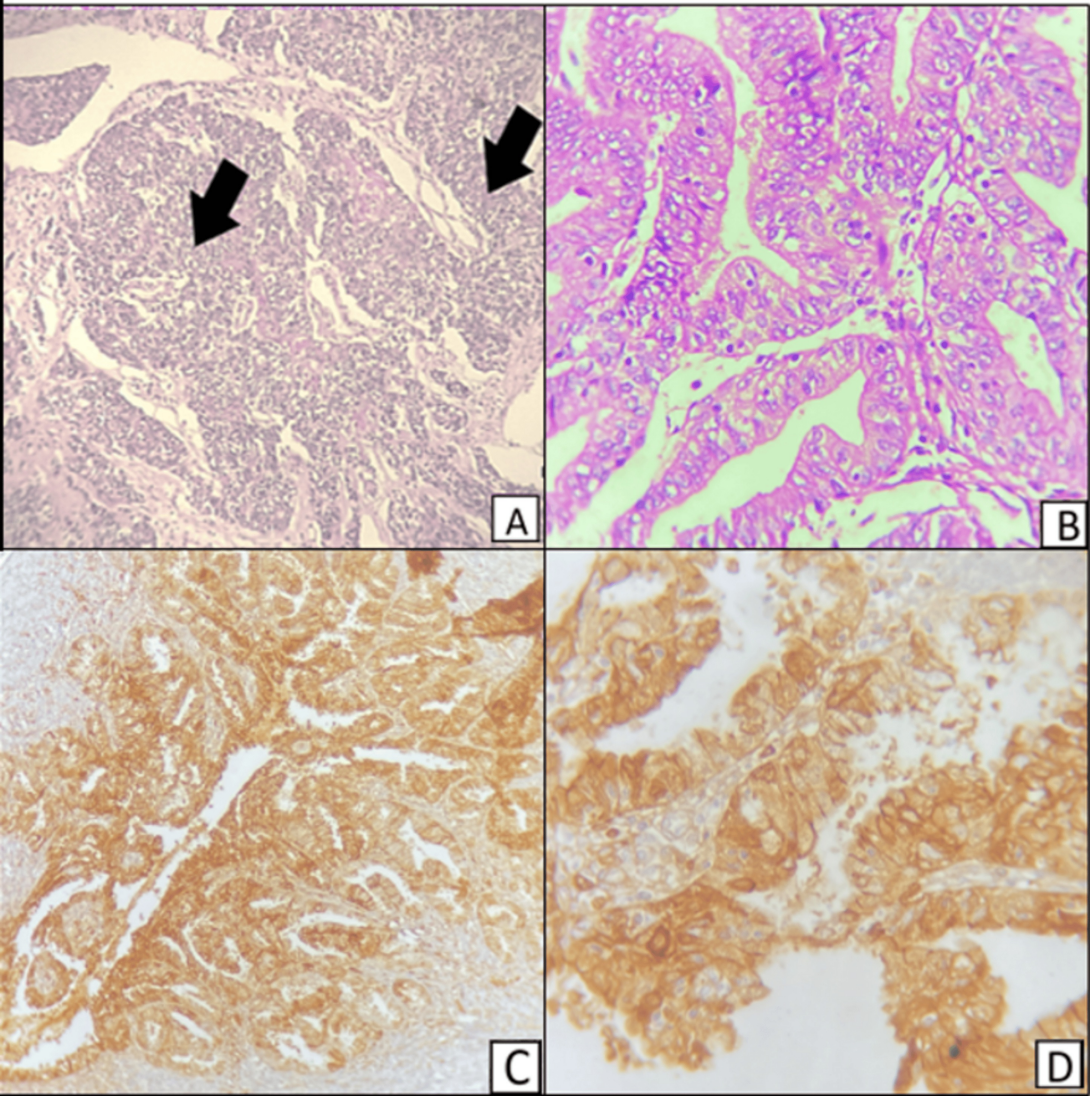
A,B - Hematoxylin and eosin stained sections; C,D - immunohistochemical-stained sections of dedifferentiated carcinoma of the endometrium A (H&E 100X) and B (H&E 400X) show dedifferentiated carcinoma of the endometrium, dedifferentiated areas pointed by black arrows; C (E-cadherin, 100X) shows diffuse cytoplasmic positive staining; D (E-cadherin, 400X) shows diffuse cytoplasmic positive staining of the endometrial glands

Figure 8 shows dedifferentiated carcinoma of the endometrium showing a dedifferentiated component in hematoxylin and eosin-stained sections. The immunohistochemical staining for E-cadherin shows diffuse positive cytoplasmic staining.

## Discussion

Cell adhesion plays a critical role in preserving the normal structural and functional properties of various cell types, including epithelial, endothelial, and neural tissues [2]. This adhesion is facilitated by a family of cell adhesion molecules known as cadherins [2,5]. By regulating the adhesion between cells, cadherins ensure the structural integrity of epithelial tissues, thereby contributing to the maintenance of healthy tissue architecture [2,5]. There are over 100 cadherins, which are classified into two broad categories: classical and non-classical cadherins [2]. Among the most well-known of these are E-cadherin and N-cadherin, which interact homotypically, meaning that they bind to the same type of cadherin on adjacent cells, forming strong adhesion bonds [5,6]. This adhesion process is calcium-dependent, requiring the presence of calcium ions to mediate the interactions between cadherins [6].

E-cadherin is a transmembrane protein situated on the cell membrane, playing a crucial role in linking the internal structure of the cell to its external environment [2,5]. It connects to the actin cytoskeleton inside the cell via several cytoplasmic domains, including α-catenin, β-catenin, and γ-catenin, forming a cadherin-catenin complex [2,6]. This complex is essential for maintaining the normal epithelial lining and the overall structural integrity of tissues. Studies have indicated that a reduction in E-cadherin expression is commonly seen in malignant tumors and is often associated with disease progression [2,6]. This downregulation is linked to the invasive and metastatic behavior of various cancers, including those in the breast, prostate, gastric, and thyroid tissues. In such cases, the loss of E-cadherin expression is frequently related to poorer prognosis and decreased survival rates [5,6]. Moreover, similar alterations in the cadherin-catenin complex have been documented in cancers of the cervix, endometrium, and ovary.

Although the specific role of E-cadherin in endometrial cancer is not completely understood, several studies suggest that methylation of the E-cadherin gene, along with genes for estrogen receptors (ER) and progesterone receptors (PR), may be involved in the early stages of endometrial cancer development [6,7]. There is substantial evidence in the literature indicating a strong correlation between increased methylation of these gene promoters and the progression of malignant lesions [6-8]. Additionally, research has examined E-cadherin expression in normal endometrial tissue during various phases of the menstrual cycle, including the proliferative, secretory, and atrophic phases [7,8]. These studies have shown that E-cadherin typically exhibits a uniform expression pattern, with strong immunostaining at cell borders and only weak staining within the cytoplasm [7,8]. Importantly, no significant differences in E-cadherin expression have been found between the different menstrual cycle phases [9]. In postmenopausal women with atrophic endometrium, a similar pattern of E-cadherin expression has been observed, with uniform distribution across cases; however, 10-15% of these cases were negative for E-cadherin expression [8,9].

This study included a total of 25 cases of EC, comprising 20 cases of endometrioid EC, four cases of serous EC, and one case of dedifferentiated EC. The sample size of this study is comparable to that of a study conducted by Ahmed et al., which included 54 cases of atypical hyperplasia (AH), EC, and normal proliferative endometrium [10]. It is worth noting that our study did not involve any cases of normal endometrium. The findings of the present study indicate that the majority of EC cases were observed in women aged 41-50 years, with 48% of the cases falling within this age group. Similarly, a study by Masjeed et al. found that the maximum incidence of carcinoma of the endometrium occurred in the age group of 50-60 years [11]. This suggests that the transition from benign to malignant epithelium may occur gradually over time, with an increasing degree of abnormality [11].

Parity, or the number of times a woman has given birth, is recognized as a key risk factor for endometrial cancer. In our investigation, 100% of the patients were multiparous women, meaning they had given birth to more than one child. This finding is in agreement with the study by Lakshmi, which observed that 85.5% of cases of endometrial malignancy were seen in multiparous women [12]. A meta-analysis conducted by Katagiri et al. further supports the idea that parity may be a risk factor for endometrial cancer, suggesting that reproductive factors play a significant role in the etiology of the disease [13]. It is well understood that estrogen promotes endometrial cell proliferation and enhances mitotic activity, both of which may contribute to the development of cancer. Conversely, progestins are thought to reduce the risk of endometrial cancer by inhibiting cell proliferation and promoting cellular differentiation.

In our study, the majority of patients with EC presented with complaints of abnormal uterine bleeding (AUB). AUB was the most commonly reported symptom, affecting 32% of the cases. This finding is in line with the results of a study conducted by Lakshmi et al., which reported that abnormal uterine bleeding was the main symptom in 56% of EC cases [12]. Additionally, a study by Whitaker et al. found that abnormal uterine bleeding was the most common presentation for both EC and endometrial hyperplasia [14]. AUB is defined as bleeding that occurs between normal menstrual periods, irregular menstrual timing, or any form of bleeding in women of reproductive age. It is one of the most common symptoms for which perimenopausal women seek medical attention from gynecologists. Given the high incidence of malignancy associated with AUB, it is essential that it be thoroughly evaluated. This evaluation typically begins with a detailed medical history, physical examination, and diagnostic testing, including ultrasound and endometrial sampling.

It is well-documented that endometrial thickness tends to increase as lesions progress toward carcinoma. However, the values observed in this study did not show any statistical significance (P<0.0005), which is consistent with findings from a study done by Saccardi et al. [15]. Their research established that endometrial thickness was higher in patients with endometrial cancer compared to those with endometrial hyperplasia.

In 2014, Ahmed et al. conducted a study investigating the expression of E-cadherin and CD10 in hyperplastic and neoplastic endometrial tissues [10]. The findings showed that the mean E-cadherin histoscore was significantly higher in cases of AH compared to endometrial adenocarcinomas, indicating that E-cadherin expression is more elevated in AH than in endometrial adenocarcinomas [10]. Similarly, a 2005 study by Moreno-Bueno et al. explored the expression of E-cadherin and catenin in endometrial hyperplasia and carcinoma, revealing that, while E-cadherin levels were reduced in AH compared to normal endometrium, the decrease was less significant than in EC [16].

In our study, 52% of EC cases exhibited negative E-cadherin expression, which accounted for 13 of the 25 cases. Heterogeneous staining with a score of 1 or 2 was observed in 28% of the cases, which corresponds to 7 of the 25 cases. Only five of the 25 cases, or 20%, showed normal E-cadherin staining. These results suggest that E-cadherin expression decreases as the histological grade of the tumor worsens. According to the FIGO classification, endometrial tumors are graded based on the percentage of solid growth patterns observed within the tumor. Grade 1 tumors exhibit less than 5% solid growth in sheets without any glandular component, Grade 2 tumors show 6-50% solid patterns, and Grade 3 tumors consist of more than 50% non-glandular solid sheets of tumor cells. In Grade 1 tumors, E-cadherin downregulation was observed in only 30% of the cases, whereas it was present in 40% of Grade 2 tumors. In Grade 3 tumors; however, all cases exhibited downregulation of E-cadherin. This correlation between tumor grade and E-cadherin expression was also discussed in a 2013 study by Koyuncuoglu et al., which found that 20% of Grade 1 tumors, 40% of Grade 2 tumors, and 72% of Grade 3 tumors showed decreased E-cadherin expression [17].

In 2021, Lewczuk et al. investigated the expression of E-cadherin, N-cadherin, and P-cadherin in endometrial cancers. Their research found that E-cadherin expression decreased in tumors with higher TNM stages compared to those with lower stages [18]. Specifically, only 15% of Stage I tumors showed reduced E-cadherin expression, while Stage II had 3%, Stage III had 15%, and Stage IV had 13% [18]. These results align with a study by Sakuragi et al. Our study also reflects these findings [19]. We categorized cases into two groups based on tumor stages and observed that the higher-stage group (T3 and T4) had a greater reduction in E-cadherin expression compared to the lower-stage group (T1 and T2) [19]. Similar results were observed using FIGO staging, where tumors classified as Stages I and II were compared with those in Stages III and IV, with the latter group showing a more pronounced decrease in E-cadherin expression [19].

This study thus shows that there is a decline in the expression of the cell adhesion molecule E-cadherin in EC as the disease progresses. The study's sample size is small, with only 25 cases, which can restrict how broadly the results can be applied. More participants would be required to confirm the findings and increase the statistical power. Given that the study was carried out in a single facility, it is possible that the patient population does not accurately reflect larger demographic groups. A multi-center strategy would offer a more varied sample, which would improve the results' external validity. The lack of thorough long-term follow-up data in the study may make it more difficult to evaluate how E-cadherin expression affects long-term results. The investigation could benefit from looking at additional molecular markers or pathways connected to endometrial cancer, as the study only looks at E-cadherin expression. The selection of cases might introduce bias, particularly if certain subtypes of endometrial cancer or stages were over- or under-represented in the study sample. This could affect the applicability of the findings to the broader population. There is a need for further studies in larger populations and in correlation with other cell adhesion molecules and more clinical parameters to come to a conclusion on whether E-cadherin can be used as a prognostic tool in predicting the outcome of EC [20,21].

## Conclusions

This study has provided valuable insights into the role of E-cadherin expression in EC and its potential as a prognostic marker. Through immunohistochemical analysis, we observed that E-cadherin, which normally demonstrates strong and pure membranous expression, exhibited decreased expression in cases of early malignant transformation within the endometrial glands. This reduction in E-cadherin expression likely results from the impairment of its intercellular adhesion function, which is crucial for maintaining tissue integrity. Notably, our findings align with previous studies that have shown that the disruption of E-cadherin expression occurs early in the progression from EH to carcinoma, suggesting that alterations in E-cadherin could be a significant marker of malignancy. The early downregulation of E-cadherin in precursor lesions such as EH highlights its potential utility in predicting the transformation of benign hyperplastic lesions into malignant ones. Furthermore, the correlation between E-cadherin expression and tumor progression underscores the importance of further research to explore whether E-cadherin could serve as a dependable marker to assess the metastatic potential of EC. Such investigations could enhance our understanding of the molecular mechanisms underlying tumor progression and aid in the development of more targeted and effective therapeutic strategies. Moreover, the study underscores the significance of evaluating cell adhesion molecules, such as E-cadherin, in conjunction with clinical and pathological features to improve early prediction and treatment of EC. As the tumor progresses from hyperplasia to carcinoma, the heterogeneity and reduction in E-cadherin expression may serve as an important biomarker for prognosis, ultimately aiding in the prompt and precise management of patients at risk for developing EC.
